# Scientific Prospects for Cannabis-Microbiome Research to Ensure Quality and Safety of Products

**DOI:** 10.3390/microorganisms8020290

**Published:** 2020-02-20

**Authors:** Vladimir Vujanovic, Darren R. Korber, Silva Vujanovic, Josko Vujanovic, Suha Jabaji

**Affiliations:** 1 Food and Bioproduct Sciences, University of Saskatchewan, Saskatoon, SK S7N 5A8, Canada; darren.korber@usask.ca; 2 Hospital Pharmacy, CISSS des Laurentides and Université de Montréal-Montreal, QC J8H 4C7, Canada; silva.vujan@gmail.com; 3 Medical Imaging, CISSS-Laurentides, Lachute, QC J8H 4C7, Canada; josko.vujanovic.1@gmail.com; 4 Plant Science, McGill University, Ste-Anne-de-Bellevue, QC H9X 3V9, Canada; suha.jabaji@mcgill.ca

**Keywords:** *Cannabis*, microbiome, NCBI and USDA databases, omics, risk management, safety

## Abstract

*Cannabis* legalization has occurred in several countries worldwide. Along with steadily growing research in *Cannabis* healthcare science, there is an increasing interest for scientific-based knowledge in plant microbiology and food science, with work connecting the plant microbiome and plant health to product quality across the value chain of cannabis. This review paper provides an overview of the state of knowledge and challenges in *Cannabis* science, and thereby identifies critical risk management and safety issues in order to capitalize on innovations while ensuring product quality control. It highlights scientific gap areas to steer future research, with an emphasis on plant-microbiome sciences committed to using cutting-edge technologies for more efficient *Cannabis* production and high-quality products intended for recreational, pharmaceutical, and medicinal use.

## 1. Introduction

The course of *Cannabis* science has been a top-down process, with the effects of the plant on humans and animals being tested before conducting extensive agronomic and ecological studies. Hence, there is a growing interest for acquiring evidence-based knowledge via integrated and multiplatform Omics-studies on *Cannabis*. In this regard, systems biology [1,2] is well-equipped to integrate findings and define the genetic variability and functional metabolites [3] in a model plant system. Model plant research, such as that of well-known *Arabidopsis thaliana* [4,5,6,7] and the extensively-studied medicinal plant, *Papaver somniferum* [8,9,10,11,12], highlight the significance of gathering information about regulation of plant fitness and homeostatic mechanisms of metabolites [13] orchestrated by the host genome and its affiliated microbial metagenome [14]. Such an approach notably reveals the importance of the plant’s genome-microbiome interactions [15], which changes under stress or disease conditions [16,17] as plants selectively source their microbiomes to suit their needs [18,19]. Similar advancement and in-depth research is required for *Cannabis*. Understanding cultivar-specificity in the *Cannabis* microbiome [20] and its fluctuating microbial phylogenetic and functional composition [21] throughout cultivation regions remains a challenge especially with climate change. Next-generation sequencing (NGS) used in model plant studies has confirmed the complex interplay between plants and their microbial communities, also known as the hologenome [22], across indoor or outdoor ecosystems [23,24]. Still, extensive and rigorous plant model studies on *Cannabis* are lacking from the perspective of improving the quality and safety of products, particularly those concerning the microbiome and pathogenic pre- and post-harvest contaminants.

## 2. Why Advance *Cannabis* Science?

Compared to the *Arabidopsis* (dicot) plant model system, the NCBI-DNA/RNA sequence data related to the microbiome of *Cannabis* (dicot) showed 429,013 vs. 22,822 bacterial 16S, as well as 5232 vs. 182 fungal ITS sequences for *Arabidopsis* and *Cannabis,* respectively (NCBI, data retrieved on 12 January 2019). Moreover, most current data on *Cannabis* come from conferences—an amalgam of scarce and heterogenous data, with only few publications raising concern about mycotoxins in *Cannabis* plants and related products [25,26]. The current state in *Cannabis* data is incomplete in addressing the potential for beneficial biocontrol [27] and plant growth-promoting [28] agents such as endorhiza endophytes [20,29] as environmental tools for combating disease-causing pathogens and mycotoxigenic molds, and far from elucidating shifts in plant biochemical traits improved by the microbiome. Consequently, model plant research, including host-microbiome partnership and interactome [30], is sorely needed in *Cannabis* science to ensure product safety and sustainability.

## 3. Where are the Opportunities for *Cannabis* Science?

**Figure 1:**
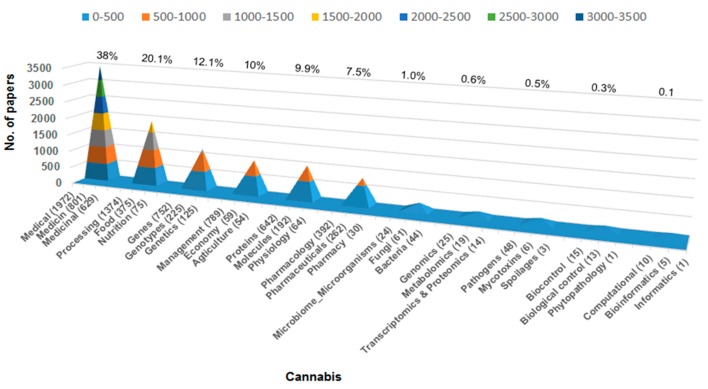
Web of Science data depicting published (1900–2018) articles on *Cannabis* related to major scientific domains (Data retrieved on 12 January 2019).

Regrouping research domains and measuring publication metrics within *Cannabis* science (Figure 1) is a necessary step in identifying knowledge gaps that need to be filled in order to advance this nascent discipline. Currently, there are a total of about 10,000 publications on *Cannabis* in the scientific literature. Web of Science data (1900–2018) suggest that medicine and food-processing topics largely prevail, together encompassing more than 50% of the published reports. Medical imaging comprises ~5% of publications, with emphasis on magnetic resonance imaging (MRI) results in the context of cannabis-related health issues [31]. Genetics, pharmaceuticals, and management represent ~40% of all cannabis literature. In contrast, reports on plant-microbiome science using Omics-driven approaches (i.e., genomics, transcriptomics, proteomics, or metabolomics) on *Cannabis* are scarce [32], representing a mere ~0.6% of published papers. 

This indicates that scientific direction of studies on *Cannabis* should include a greater emphasis on Omics-based approaches (using the *Arabidopsis* and/or medicinal plant model as a benchmark). A call to increase knowledge in these key areas is essential to promote research on *Cannabis*-pathogen and *Cannabis*-beneficial organism interactions, and would provide regulators of the *Cannabis* industry with science-based data necessary for making rational decisions and sound legislation. Although such research is emerging in some countries (e.g., Canada, Mexico, South Africa, Uruguay, and US), it needs to gain more momentum. 

## 4. What are the Major Risks Associated with *Cannabis*?

According to Web of Science data (2019), less than 1% of published articles refer to *Cannabis*’ microbiome (fungi and bacteria); whereas pathogens, mycotoxins, and product spoilage organisms represent a mere 0.5% of the total number of papers (Figure 1). Phytopathology, including biological control and decomposition of toxins, is also under-represented at 0.3% of the total. Similarly, computational and bioinformatics disciplines [33] that can generate/process large biological data sets and help advance interdisciplinary *Cannabis* biosciences are severely under-utilized (0.1%). These results reveal significant risks in terms of product quality and safety. Furthermore, the presence of pathogens and mycotoxins are expected to rise together with increased demands for *Cannabis* production at a large scale, under both greenhouse and field conditions. The problem related to specific molds (i.e., *Aspergillus* and *Penicillium*) and high concentration of mycotoxins and toxic pesticide residues is amplified by greenhouse/closed and relatively humid environmental conditions [34]. In many instances, the open agricultural fields are rather exposed to climate change-associated phytopathogenic and mycotoxigenic fungi and infectious bacteria [35,36]. In both cases and environments, unhealthy biological and chemical contaminants in *Cannabis* samples and/or commodities may induce serious physical, mental, behavioral, and social health consequences in humans, in the event that proper preventative measures are not taken. A recent UC Davis Medical Center report authored by W. Walker [37] based on DNA analyses found that >20% of tested medical *Cannabis* samples were contaminated with a range of dangerous bacteria and fungal molds. Thus, even *Cannabis* labeled as medicinal-grade could pose a dangerous and potentially lethal threat to human beings, especially in vulnerable population sub-groups with suppressed immune systems (e.g., in context of cancer, transplant recipients, and HIV/AIDS) or in the elderly, where *Cannabis* is increasingly used for pain/mood management and for inducing sleep [38,39]. Thus, quality research on *Cannabis* in the healthcare sector is intricately linked to research knowledge that ensures health, quality, and safety of *Cannabis* plants and the production environment.

## 5. What are the Sources of Host Infection and Diminished Quality Versus Safety of *Cannabis* Products?

A total of 305 literature records for 105 fungal taxa were found in the USDA-Fungi database using the criteria according to Farr et al. [40]: Host Genus = Cannabis. The USDA data and scientific information on *Cannabis*-fungus relationship [40] have been obtained by culture and culture- independent methods and published in peer-reviewed articles and research reports of the governmental plant pathology laboratories worldwide. The pathobiota listed in Table S1 were reported on *Cannabis* host, and led to disease symptoms on different host organs according to the American Phytopathological Society (https://www.apsnet.org/publications/Pages/default.aspx) and Web of Science (https://clarivate.com/webofsciencegroup/solutions/web-of-science/) publications. Most of the documented phytopathogens (Table S1) broadly-include the fungal genera *Alternaria, Ascohyta/Phoma, Botrytis, Oidium, -, Sclerotinia, Septoria, Thanatephorus/Rhizoctonia,* and *Verticillium*, and the chromistan or protozoan fungal genera *Pythium* and *Phytophthora* to name a few. Host-specific pathogens, such as *Cercopsora cannabina*, have also been reported in agricultural regions worldwide, including Eurasia (e.g., Cambodia, China, India, Pakistan, and Russia), Africa (e.g., Uganda), and North America (e.g., Mississippi and Wisconsin). Moreover, occurrence of fungal pathobiota, such as *Fusaria* on *Cannabis sativa* host, shows certain site-specificity, as noted in the United States: California—*F. brachygibbosum, F. equiseti, F. radicicola, F. oxysporum,* and *F. solani*; Illinois—*F. oxysporum* f. sp., *cannabis F.* sp., *F. solani*; and Virginia—*F. sulphureum*. In addition, similar distribution patterns linked with this plant’s production system have been detected on a worldwide scale: For example, *Achlya aquatica* as a water mold—India; *Phymatotrichum* sp. causing root rot—Mexico; *Pythium* spp. inducing damping off— Canada and United States; *Pleosphaerulina cannabina* causing pepper spot—USSR/Russia; *Pseudoperonospora cannabina* responsible for downy mildew—Poland; *Puccinia cynodontis* causing rust and *Verticillium albo-atrum* wilt—China; *Sclerotium bataticola* responsible for charcoal rot—Bulgaria; and *Ramularia collo-cygni* causing leaf spot—Europe. These examples highlight the importance of preventing spread of unwanted fungal species in emerging *Cannabis* growing regions [41], and stress the need to reinforce quarantine assessment and control measures related to *Cannabis* trade. 

From an economic standpoint, *Botrytis cinerea* and *Erysiphe* species complex are currently the most widespread pathogens of *Cannabis* worldwide. Damaging flowering buds and stalks, *B. cinerea* causes gray mold and attacks flowers, fresh fruits, and vegetables in hundreds of other hosts, including vineyards, worldwide [42]. *B. cinerea* produces two major phytotoxins: the sesquiterpene botrydial and the polyketide botcinic acid, which are important virulence factors in *B. cinerea* [43]. The BcAtf1 gene has been reported as the global regulator of virulence, controlling various differentiation processes and phytotoxin production in *B. cinerea* [44]. Still, no effective management measure(s) exists against *B. cinerea* [41] or other *Cannabis*-associated molds due to their genomic plasticity and development of drug resistance. *Erysiphe* spp., the causal agents of powdery mildew (PM) on several cucurbits attacking leaves and buds, are obligate pathogens. There are no *Cannabis* varieties resistant to powdery mildew; however, various plant immune pathways can limit the extent of fungal invasion. Recessively inherited loss-of-function alleles of Mildew Locus O (*Mlo*) genes confer a prominent type of effective powdery mildew resistance [45]. In both instances, these two pathogens are common serious pathogens to many crops, including grapes. Hence, for example, those who (such as in Germany and France) use moldy gray grapes infected with *B. cinerea* to make “noble rot” wine and those who (such as Canada and US) employ late harvest to make “icewine” (increased risk of *B. cinerea* infection) should exert extra caution when introducing *Cannabis* into regional agronomic production systems. Most importantly, this calls for each agricultural region to chart and address potential pathogens and risks, and establish guidelines for safe and sustainable *Cannabis* production.

## 6. Indoor Cultivation and Storage of *Cannabis* are Propitious to Fungal and Bacterial Contamination

Medical, as well as recreational *Cannabis* grown in indoor facilities is exposed to a plethora of microbial contaminants occurring on pre- and post-harvest *Cannabis* inflorescence buds. The literature addresses an extensive list of fungal and bacterial contaminants, as well as of associated toxins, on *Cannabis* [20,46,47,48]. Taken together, published results confirm that viable organisms, including those that produce mycotoxins and endotoxins, can be recovered from *Cannabis*, potentially posing a serious risk to immunosuppressed individuals [48]. Monitoring studies examining pathogen and mold spore levels within cannabis growing facilities would provide useful insights into the diversity and changes that occur in these populations. 

Medicinal and recreational *Cannabis* in Canada, as well as in certain jurisdictions in the US under the Access to *Cannabis* for Medical Purposes Regulations (ACMPR), require microbial load testing. Acceptable microbial loads, expressed as colony forming units (CFU) per gram or millilitre, must be less than 10,000; values higher than the acceptable level are an indication of poor curing and handling practices. However, being below the cut-off CFU values does not mean that the tested product contains no endotoxins or mycotoxins. Furthermore, while desiccation of the flowers and high temperature would be expected to decrease viable microbial counts, it would not eliminate all of the microorganisms nor the endotoxins and mycotoxins that they would produce (or have already produced). Gamma irradiation, where material is exposed to a high powered gamma radiation source, is another approved method by Health Canada (https://www.canada.ca/) for decontamination which does not cause changes in the content of THC and CBD but does alter terpene quality slightly [49]. The amount of gamma radiation used is calibrated to be sufficient to cause enough DNA damage such that no cells remain viable. As with high temperature drying, this treatment would reduce viable microbial counts, but would not eliminate the dead remains of the microbes nor any endotoxins and mycotoxins that were already present. Other methods, such as cold plasma sterilization, attempt to attain an optimal balance between product activity and safety [39]. Taken together, there is an urgent need to develop and validate different effective methods for reducing the microbial load in medicinal and recreational *Cannabis* to reduce the risk of fatal opportunistic infections associated with *Cannabis* among patients or susceptible individuals. 

*Cannabis* inflorescence buds are often contaminated with molds and mycotoxins [46], particularly if not stored properly [50]. Methods for controlling mycotoxins are mostly preventive during production, handling, transportation, storage, and processing [51,52]. Two of the main types of mycotoxins associated with contaminated cannabis products are aflatoxins and ochratoxins (produced by *Aspergillus flavus, A. fumigatus, A. niger,* and *A. terreus*). Smoked marijuana contaminated with aspergilla have developed clinical, laboratory, and radiologic findings consistent with invasive pulmonary—and allergic bronchopulmonary aspergillosis [53,54]. In addition, the accumulation of aflatoxins can also cause lung and liver cancer [55] and can cross the placental barrier to exert harmful effects in the fetus [56]. Ochratoxins, such as those produced by *Aspergillus ochraceous,* have a similar mutagenic and carcinogenic profile to aflatoxins. Aflatoxins, but not fumonisins (produced by *Fusarium* species) nor ochratoxin A, are tested under the Canadian ACMPR program. While full mycotoxin testing of *Cannabis* is possible, thresholds for toxicity have not been established [57], representing future challenges for cannabis and cannabinoid sciences.

*Cannabis* extracts and concentrates require different types of microbial screening than that conduced on dried flower materials. The process of extracting cannabinoids with solvents most likely will sterilize the product, but more data is required to prove that this is the case. A real danger is that spores of mycotoxigenic fungi, such as *Aspergillus* and *Fusarium,* could survive the extraction process [58]. For example, aflatoxins—AFBs (AFB1 and AFB2s) and fumonisins—FUMs (FB1 and FB2) are all very highly soluble in the same solvents that are used to concentrate cannabinoids from *Cannabis* plant material [51]. Thus, as the cannabinoids are concentrated into waxes and oils, so too would be any mycotoxins that are present. In this case, *Aspergillus* testing would be needed on such extracts, mainly if they were destined for smoking or vaporization [59]. Moreover, recent findings suggest that intestinal microbiota have profound interactions, affecting gut, as well as overall health. Ingested FUM and AFB mycotoxins reportedly induce a gut microbiome shift in a dose-dependent manner (e.g., an increased number of *Clostridiales* and *Bacteroidales* vs. a decrease in the number of *Lactobacillales* from *Firmicutes*, *Streptococcus* sp., and *Lactococcus* sp., respectively) [21], thus altering the normal gut equilibrium. 

If *Cannabis* products were not consumed by smoking, the existing guidelines for pharmaceuticals or agricultural products would apply. The only potential source of safety regulations pertaining to plant material absorbed by inhalation would be the tobacco industry—though that industry does not publish such information and has only recently been subject to federal oversight in response to findings. Notably, some peer-reviewed articles have established a direct link between tobacco cigarette products and contamination with mycotoxigenic producing fungi and endotoxic bacteria, leading to chronic tissue inflammation of the mouth and lungs [48,60]. Further, in a single comparative study between cultured marijuana and tobacco mold loads, an indicative difference of 100,000 CFU of mold per gram of marijuana vs. 200 CFU of mold per gram of tobacco were registered, respectively [61,62]. Recommended acceptable thresholds <200 CFU/g have been suggested for microbiological (Shiga-toxin producing *Escherichia coli*, *Salmonella* spp., and *Aspergillus* spp. contaminants in marijuana products [57]. Similarly, as reported for marijuana [47], >90% of the tobacco cigarette samples were contaminated with Gram-positive and Gram-negative endotoxic bacteria, including *Acinetobacter, Bacillus, Burkholderia, Clostridium, Klebsiella, Pseudomonas aeruginosa*, and *Serratia* [62,63]. It also seems that smoking *Cannabis* and tobacco, including e-cigarettes device delivery, can increase both fungal and bacterial toxic contaminants to daily cigarette users, together with pesticides, molds, bacteria, metals, and solvents [64,65].

## 7. Risks and Effects of Consumption of Contaminated *Cannabis*

Case reports [66,67,68,69,70] describing the effect of smoking, vaping, or inhaling aerosolized contaminated marijuana demonstrate some of the graver risks to patients, especially those with leukemia, lymphoma, AIDS, or those with medical conditions requiring immune-suppressing therapies. However, the disease has also been noted in non-immunocompromised patients, albeit at a less frequent rate. The associated range of disease is wide, from cryptococcal meningitis to invasive or lung aspergillosis to fungal sinusitis [58,71,72,73,74,75]. Together, these cases highlight the potential risks associated with smoking marijuana. Understanding the full health burden associated with marijuana is hindered by a lack of disclosure by patients. It is important to stress that legal cannabis products [76] are not void of contamination and health risks. 

Marijuana vaping devices and products account for 30% of the *Cannabis* industry in the USA. Vaping devices are appealing to marijuana smokers because they do not require joint rolling, are discreet, leave no traces of ash and have little smell. A recent [77] outbreak of vaping-related lung illnesses in California resulting in several deaths due to vaped-THC has experts and officials at the Centre for Disease Control and Prevention worrying about their risks, and the potentially dangerous consequences are only now becoming evident. Recent findings further explored a potential risk of microbiologically contaminated cannabis smoking related to THC compound [78]. Apparently, the THC weakened the immune system and reduced the ability of T-cells and alveolar macrophages to protect the body from foreign pathogens, thus lowering defense against infections in lung, even in healthy cannabis smokers [79,80,81]. A weakened immune response in the lung predisposes cannabis smokers to affliction by viral, bacterial, or fungal pathogens that would typically pose little threat to a healthy immune system [81]. Fungal contamination has been highlighted in several case reports of lung infections, including from *Aspergillus*, which frequently occurs on *Cannabis* plants [67,82].

Potential harm might also arise from the consumption of bacterially contaminated *Cannabis* products, including those containing *Salmonella enteritis*, indicating that oral use may also bear health and safety issues [83]. These harms come in addition to the potentially inherent injury from *Cannabis*, for example on the respiratory system. *Cannabis* smoking is known to affect alveolar macrophages [84], cilia, and mucus-secreting cells of the respiratory system [85], thereby increasing risk of pulmonary infection. Little is known on whether smoking devices help mitigate this problem: vaping is associated with several issues, whereas pipe-smoking does not seem to be protective against *Cannabis* yeast contaminants, notably [65,86]. There also appears to be a baseline sensitization to *Aspergillus* in *Cannabis* smokers [38,87]. Thus, as mentioned by the Cannabis Advisory Panel and Working Group of the Association of Official Analytical Chemists (AOAC) [65] “new sets of standards and best practices to help guide regulators and the industry toward a more cohesive, empirical, and science-based approach” are needed. In June 2019, Illinois became the 11th US state—plus the District of Columbia—to legalize recreational cannabis sale or use.

## 8. Where do the Scientific Solutions Lie?

Characterization, prevention, and control of *Cannabis* pathogens, including pre- and post-harvest molds and their mycotoxins, will represent a major challenge for *Cannabis* science and safe product production. Preventive research on the photobiology of indoor production [88] can address common pathobiology problems across *Cannabis* growth stages under LED and HPS lighting systems. Associated meta-analyses [89] can further predict *Cannabis* yield or determine the mycotoxin profile in diseased plants under light stress. Contamination of *Cannabis* plants and products (i.e., recreational- and pharmaceutical-grades) with mycotoxigenic organisms, including species of *Aspergillus, Penicillium,* and *Fusarium,* pose serious challenges [90]. Intensive research will be required to determine at what stage of the *Cannabis* production chain (harvesting, drying, storage, processing) and for which active pharmaceutical ingredient (API) [91] these fungi/molds contribute to product contamination, and what measure should be taken to minimize contamination. Culture-dependent and -independent methods, coupled with quantitative measurements of mycotoxins and their encoding genes, are urgently required to assess the bioload at every stage of *Cannabis* production chain.

The diversity of *Cannabis*-associated *Aspergillus, Cladosporium, Fusarium, Mucor,* and *Penicillium* molds (Table S1), as well as their harmful toxins (e.g., aflatoxins, fumonisins, ochratoxins, trichothecenes, and T-2 toxins), are but the tip of the iceberg. These molds are spore-producing generalists, and can spread by air as a vehicle with significant potential for distribution across production regions and continents. *Aspergillus* is a mold that produces extremely hardy spores and is capable of rapid replication (*r*-strategists) at much lower water and nutrient levels than most microorganisms. *A. flavus*, *A. niger,* and *A. parasiticus* are molds linked to the *Cannabis* host (Table S1) that may be particularly hazardous to asthma patients. According to Holmes et al. [92], gardeners and farmers in particular are believed to inhale thousands of *Aspergilli* spores every day. It is noteworthy that pulmonary aspergillosis can be hard to diagnose and treat—especially invasive aspergillosis, for which the mortality rate is quite high, which calls for preventing the establishment of molds in *Cannabis* production systems. 

**Figure 2:**
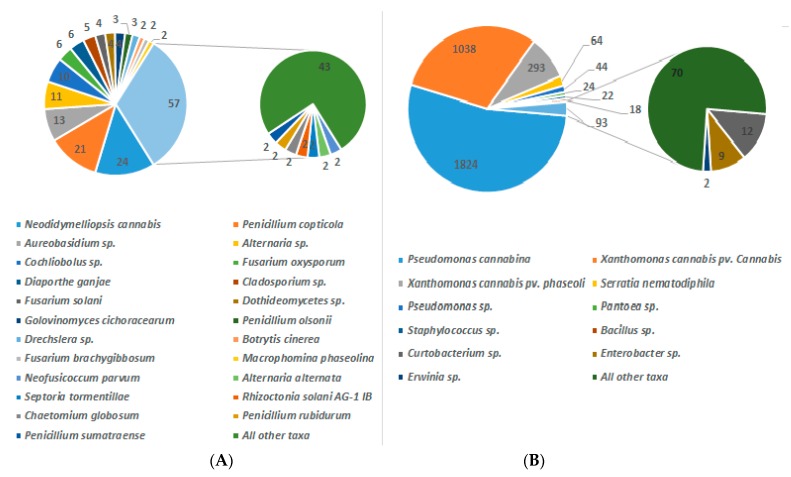
Diversity of *Cannabis* fungal taxa based on NCBI database: ≥2 ITS (internal transcribed spacer) rRNA sequences in NCBI (**A**) left panel; diversity of *Cannabis* bacterial taxa based on NCBI: ≥2 16S rRNA sequences (**B**) right panel (Data retrieved on 12 January 2019).

The USDA list of fungi (Table S1) indicates that *Cannabis* is also host for *Trichoderma* and *Trichothecium (T. roseum)*, potential biological control agents (BCA) [93]. However, such generalists also pose important health risks [94]. Indeed, BCA based on *Trichoderma, Trichothecium, Myrothecium,* and *Stachybotrys* produce trichothecenes, which are known mycotoxins. A single BCA generalist— such as *Trichoderma viride*, a necrotrophic mycoparasite, can also be the source of several mycotoxins: gliotoxin, T-2 toxin, trichodermin, trichodermol, and viridiol [95]. In Canada, under the Access to Cannabis for Medical Purposes Regulations (ACMPR; https://www.canada.ca/en/health-canada/services/cannabis-regulations-licensed-producers/pest-control-products.html), licensed producers may only select from 22 pest-control products that are currently approved for use on cannabis under the Pest Control Products Act (PCPA). Among them is Rootshield (*Trichoderma harzianum*). Thus, fungal disease control options are currently limited: chemicals are controversial, while safe BCA are yet to be determined. This situation highlights the need for further research to find better BCA, based on specific-biotrophic mycoparasites—such as *Sphaerodes* against *Fusaria* and *Ampelomyces* against *Erysiphales*—to prevent and control plant diseases and mycotoxin accumulation [94]. It is of particular interest to select safe biocontrol candidates as public perception goes in support of eco-friendly BCA products against plant pests [96]. To achieve this goal, our overall knowledge of *Cannabis* pathobiota must rapidly advance to include both fungi and bacteria. For instance, the NCBI DNA/RNA data should better concord with taxonomical fungal identifications in the USDA database. In addition, an organism’s biogeographical distribution should be more accurately related to genomic sequences. At the moment, a comprehensive NCBI list of fungal taxa on *Cannabis* shows 22 species with ≥2 ITS rRNA sequences deposited (Figure 2A), which is low compared to the USDA record of 105 species (Table S1). The NCBI list predominantly reports the recently discovered *Neodidymelliopsis cannabis* [97], a cause of black stem canker [98]. 

The NCBI also lists 12 bacteria found on *Cannabis,* based on 16S rRNA (Figure 2B), illustrating a predominance of *Pseudomonas cannabina,* a cause of leaf and stem rot in *Cannabis* [99,100]. This *Pseudomonas* species contains several virulent pathovars [101], which merits further investigation. The 16S data also includes human (e.g., *Staphylococcus*, *Enterobacter*, and *Serrata*) and other plant (*Erwinia* and *Xanthomonas*) pathogens, potential biocontrol (e.g., *Bacillus*) and PGP (plant growth promoting, e.g., *Pantoea*) agents. 

Rare metagenomic and qPCR studies [102] in medical *Cannabis* literature reveal the existence of additional pathogenic or toxigenic bacterial and fungal species. Accordingly, *Acinetobacter baumannii, Clostridium botulinum*, *Escherichia coli, Pseudomonas aeruginosa, Ralstonia pickettii, Salmonella enterica, Stenotrophomonas maltophilia, Aspergillus ostianus, A. sydowii, Penicillium citrinum,* and *P. steckii* have been detected. An increasing number of new reports also identify *Golovinomyces spadiceus* powdery mildew and *Cercospora* cf. *flagellaris* leaf spot on industrial *Cannabis* in the US [103,104], suggesting the importance of employing more advanced high-throughput technologies in *Cannabis* research.

## 9. What are The Future Technological Advancements?

Continued advances in high-throughput next-generation (NGS) sequencing make microbiome and Omics-based studies increasingly accessible [105,106]. Implementation or adaptation of surveillance networks, such as Food-Net (Foodborne Diseases Active Surveillance Network; https://www.cdc.gov/foodnet/index.html) and PulseNet (https://www.cdc.gov/pulsenet/index.html), could be of value to *Cannabis* production venues, as they would allow tracking of region-specific susceptibility to microbial (notably fungal or bacterial) outbreaks using Omics characterized by big data production. This promises more efficient surveillance, plant disease and mold contamination control, and global prevention systems. Improved antimicrobial BCA cells, bioactive (e.g., essential oil and terpene) molecules [107,108], and silver nanoparticles [109] seem plausible control agents, often with added antioxidant benefit [110], to be considered in *Cannabis* production. Synthetic biologists have seen beneficial fungal yeasts and bacteria cells as “green” factories to produce cannabinoids [111]. In addition to preventing contamination, this could allow for novel cannabinoids to be synthesized. The growing knowledge of CRISPR/Cas9-mediated genome-editing technologies has further revolutionized genetic studies in a wide range of organisms [112] and is expected to be increasingly employed to improve *Cannabis* plant health, quality, and safety of products. One aim could be to enhance human immunity to *Cannabis*-transmitted toxic substances and pathogenic diseases. Further, human gene-expression studies (nutrigenomics) should be linked to shifts in *Cannabis* metabolomics induced by microbiomes across production environments; this could also play a vital role in determining the wellness or disease status of the human body [113]. Once achieved, the integration of data from different Omics-Cas9 approaches will improve the diagnosis, monitoring and therapy of diseases by allowing the identification of novel potentially-actionable biomarkers in view of personalized medicine [105]. Such information would be of great interest, given the increasing popularity of microgrowery (https://www.canada.ca/en/services/health/campaigns/cannabis/proposed-requirements-cultivation-processing-federal-sale-licences.html), indoor and outdoor/field *Cannabis* cultivation, and production through licensing.

## 10. Conclusions and Future Prospects

The emerging *Cannabis* production sector currently lacks the strong foundational science and knowledge-based, public-domain information necessary to ensure the health of consumers and well-being of society. Very little is known about *Cannabis* genetics and breeding and how the microbiome offers critical genetic variability to *Cannabis* that could lead to new approaches for *Cannabis* breeding strategies, product safety and quality, whether intended for recreational, pharmaceutical, or medicinal purposes. In particular, information about the microbiome of *Cannabis* is scarce, and security concerns related to human health and *Cannabis* production sustainability have yet to be addressed, particularly with respect to pathogenic microbiomes, molds, and mycotoxins. 

At the same time, beneficial biocontrol and plant growth-promoting microbiomes represent promising tools to tackle diseases and mycotoxins, as well as to improve plant biochemical traits. Increased NCBI data on *Cannabis*-associated microbiomes, coupled with enhanced metagenomics and Omics datasets, bioinformatics, and plant phenotyping and imaging, appear essential steps to improve *Cannabis* agriculture risk management, as well as product security, safety, and quality. In addition, microbiome-based synthetic biology offers further advantages of preventing contamination and helping in uncovering new Cannabinoids. Such research would have important implications for *Cannabis* production, both in terms of industrial quality testing and food safety. 

How can system biology science and technologies keep up the pace with the dynamic *Cannabis* industry? *Cannabis* science, based on the principle of healthy plants and high safety standards to control microbial contaminants throughout the value chain, can prevent future biological hazards and make quality products more predictable for improved health care and safe *Cannabis* consumption. 

## Supplementary Materials

Supplementary materials can be found at https://www.mdpi.com/2076-2607/8/2/290/s1. Table S1: Facts about fungal diseases and molds as potential risks for *Cannabis sativa* product quality and safety for human health.
