# Supplementary material for: Scientific Prospects for Cannabis-Microbiome Research to Ensure Quality and Safety of Products
Source: Microorganisms. 2020 Feb 20;8(2):290. doi: 10.3390/microorganisms8020290 (PMC7074860; doi:10.3390/microorganisms8020290)
Supplement: Supplementary file 1 [file microorganisms-08-00290-s001.pdf]

Supplement: Table S1. Facts about fungal diseases and molds as potential risk for *Cannabis sativa* product quality and safety for human health

Host	Fungus*	Risk or Potential Threat**	Origin
Cannabis sativa	Achlya aquatica	Damping off	India
	Achlya sp.	Damping off	Alabama
	Alternaria alternata	Brown blight, Mold	India
	Alternaria porri	Unknown	France
	Alternaria sp.	Unknown	Manitoba, Iowa
	Alternaria tenuis	Mold	Kyrgyzstan, Ilinois, Kanzas
	Ascochyta boehmeriae	Leaf blight	China
	Ascochyta prasadii	Leaf blight	China
	Ascochyta sp.	Leaf blight	China
	Aspergillus flavus	Mold, Myxotoxins	Maryland, Virginia, Wisconsin
	Aspergillus niger	Mold, Myxotoxins	Maryland, Wisconsin
	Aspergillus parasiticus	Mold, Myxotoxins	Virginia
	Aspergillus sp.	Mold, Myxotoxins	Iowa
	Botryosphaeria marconii	Hemp blight	France,Italy, Lithuania,Maryland,Russia, Virginia
	Botrytis cinerea	Gray mold	Bulgaria,California, Canada, Oregon,Virginia
	Botrytis sp.	Gray mold	Canada, Manitoba
	Cephalosporium sp.	Leaf strip, wilt	Iowa
	Cercospora cannabina	Olive leaf spot	Cambodia,China, India, Mississippi, Pakistan, Uganda, Russia, Wisconsin
	Cercospora cannabis	Olive leaf spot	China,India, Japan. Missouri
	Cercospora sp.	Olive leaf spot	Cambodia
	Chaetomium succineum	Unknown	India
	Cladosporium tenuissimum	Stem Canker, Mold	India
	Colletotrichum spp.	Anthracnose	China
	Coniothyrium sp.	Unknown	Iowa
	Corticium solani - (Rhizoctonia solani)	Rot rot, Damping off, Foliar bligh	Greece
	Curvularia cymbopogonis	Leaf spot, Blight	Nepal
	Cylindrosporium sp.	Blight	Korea, Maryland
	Dendrophoma marconii (Botryosphaeria marconii)	Twig blight	Chile
	Diaporthe arctii var. achilleae	Stem canker	Italy
	Diaporthe ganjae	Stem canker	Illinois, United States
	Didymella arcuata	Leaf Blight	Germany
	Diplodiella ramentacea	Unknown	Poland
	Epicoccum spp.	Black dot	India, Iowa
	Fusarium avenaceum var. herbarum	Stem Canker	Poland
	Fusarium brachygibbosum:	Unknown	California
	Fusarium equiseti	Bud rot	California
	Fusarium radicicola	unknown	California, Canada, Poland
	Fusarium lateritium	Stem canker	Poland
	Fusarium oxysporum	Wilt, Bud rot, Damping off	California, Canada China,Poland
	Fusarium oxysporum f. sp. cannabis	Wilt, Bud rot	California, Ilinois
	Fusarium solani	Foot rot, Root rot and Bud rot	California,Canada, Poland
	Fusarium sp.	Unknown	Canada,Illinois, Indiana, Iowa, Mexico, Russia, Virginia, Wisconsin
	Fusarium sulphureum	Damping off, Stem canker	Virginia
	Glomus mosseae	Root mycorrhiza	Illinois, United States
	Golovinomyces cichoracearum	Powdery Mildew	Canada
	Helicomina cannabis	Olive leaf spot	India
	Hormodendrum sp.	Mold	Iowa
	Hypomyces cancri - (Nectria cancri)	Foot rot	Maryland
	Leptomitus lacteus	Seed mold	Poland
	Leptosphaeria spp.	Blight	Italy, Romania
	Leptosphaerulina trifolii	Leaf and stem spot	India
	Leveillula taurica	Powdery mildew	Asia, France, Turkey, Russia
	Macrophoma sp.	Stem canker, Wilt	Maryland, Virginia
	Macrophomina phaseolina	Charcoal Rot, Damping Off	Illinois, Iran, Spain
	Micropeltopsis cannabis	Unknown	France
	Monilia sp.	Brown rot of fruit	Iowa
	Mucor sp.	Mold	Wisconsin
	Mycosphaerella cannabis - (Neodidymelliopsis cannabis)	Stem canker	China
	Myrothecium roridum - (Paramyrothecium roridum)	Leaf spot	India
	Oidium sp.	Powdery mildew	Italy, South Africa
	Ophiobolus anguillides	Stem cancer	Minnesota
	Orbilia luteola	Unknown	France
	Papularia sphaerosperma - (Arthrinium phaeospermum)	Unknown	India
	Pellicularia rolfsii - (Athelia rolfsii)	Souhern blight	Korea
	Penicillium copticola	Bud rot, Mold	Canada
	Penicillium olsonii	Bud rot, Mold	Canada
	Penicillium sp.	Unknown	Iowa, Wisconsin
	Periconia spp.	Leaf spot	India, Iowa
	Peronoplasmopara cannabina - (Pseudoperonospora cannabina)	Downy Mildew	Central Asia, China, Lithuania, Pakistan, Poland, Russia
	Phoma cannabis - (Neodidymelliopsis cannabis)	Leaf Blight, Stem canker	Asia, Europe, United States
	Phoma herbarum	Leaf Blight, Stem canker	China
	Phoma nebulosa - (Phomatodes nebulosa)	Leaf Blight, Stem canker	Netherlands
	Phoma sp.	Leaf Blight, Stem canker	Poland, Iowa
	Phomopsis cannabina	White leaf spot, Stem canker	India, Illinois
	Phomopsis sp.	White leaf spot, Stem canker	Illinois
	Phyllosticta cannabis - (Neodidymelliopsis cannabis)	Leaf spot	Bulgaria,China, India,Wisconsin
	Phyllosticta straminella - (Macrophoma straminella)	Leaf spot	China, Korea
	Phymatotrichum omnivorum - (Phymatotrichopsis omnivora)	Root rot	Arizona, Texas
	Phymatotrichum sp.	Rot rot, Damping off, Foliar bligh	Mexico
	Phytophthora sp.	Root rot	Korea
	Pithomyces chartarum - (Pseudopithomyces chartarum)	Leaf blight	India
	Pleosphaerulina cannabina	Pepper spot	Russia
	Podosphaera macularis	Powdery mildew	Switzerland
	Pseudocercospora cannabina	Olive leaf spot	China, India, Korea,Poland
	Pseudoperonospora cannabina	Downy mildew	Andorra, Austria, Bulgaria, China, Europe, France, Hungary, Japan, Korea, Kyrgyzstan, Latvia, Poland, Portugal, Romania, Russia, Spain, Switzerland
	Puccinia cynodontis	Rust	China
	Pythium aphanidermatum	Root Rot, Damping off	California, Canada, Indiana, United States
	Pythium dissotocum	Damping off	Canada
	Pythium myriotylum	Damping off	Canada
	Pythium ultimum - (Globisporangium ultimum)	Root Rot, Damping off	Canada, Indiana
	Ramularia collo-cygni	Leaf spot	Austria, Europe
	Rhizoctonia sp.	Root Rot	Netherlands
	Schiffnerula cannabis	Black Mildew	Nepal
	Sclerotinia sclerotiorum	Hemp Canker, White mold	Canada (Manitoba, Prince Edward Island), China, France, Montana
	Sclerotium bataticola - (Macrophomina phaseolina)	Damping off, Charcoal rot, Collar rot, Stem rot, Root rot	Bulgaria
	Sclerotium rolfsii - (Athelia rolfsii)	Southern blight, Root and steam rot	Italy, South Carolina, Texas
	Septoria cannabina - (Septoria neocannabina)	Yellow leaf spot	Canada, New York, Romania
	Septoria cannabis	Yellow leaf spot	Bulgaria, ,Canada, China -, Florida, Illinois, Iowa, India, Korea, Minnesota, Poland, Romania, Russia, South Dakota,Southeastern states,Taiwan, Texas
	Sphaerotheca macularis - (Podosphaera macularis)	Powdery mildew	Illinois
	Spicaria sp.	Unknown	Iowa
	Stemphylium cannabinum	Leaf Spot, Hemp Canker	Bulgaria
	Thanatephorus cucumeris - (Rhizoctonia solani)	Sheath blight, seed and root rot	China
	Trichoderma sp.	Unknown	Iowa
	Trichothecium roseum	Pink rot, Mold	India, Iowa
	Uredo kriegeriana	Rust	Germany, Russia (USSR)
	Verticillium albo-atrum	Tracheomycosis, Wilt	China

*** Citation:** Farr, D.F., & Rossman, A.Y. Fungal Databases, U.S. National Fungus Collections, ARS, USDA. 305 records retrieved on July 7th, 2019, from https://nt.ars-grin.gov/fungaldatabases/

****Source**: An updated list of the Common Names of Plant Diseases by John Michael McPartland 2003. Diseases of Hemp. APS Publications (link: https://www.apsnet.org/publications/commonnames/Pages/Hemp.aspx) using the Web of Science (data retreived on July 21, 2019)
